# Clinical and genetic characterization of 47 Chinese pediatric patients with Pitt–Hopkins syndrome: a retrospective study

**DOI:** 10.1186/s13023-024-03055-7

**Published:** 2024-02-08

**Authors:** Tingting Zhao, Shengnan Wu, Yiping Shen, Jing Leng, Georgi Z. Genchev, Hui Lu, Jincai Feng

**Affiliations:** 1 Shanghai Engineering Research Center for Big Data in Pediatric Precision Medicine, Center for Biomedical Informatics, Shanghai Children’s Hospital, School of Medicine, Shanghai Jiao Tong University, Shanghai, China; 2 Molecular Diagnostic Laboratory, Shanghai Children’s Hospital, School of Medicine, Shanghai Jiao Tong University, Shanghai, China; 3 Division of Genetics and Genomics, Boston Children’s Hospital, Harvard Medical School, Boston, USA; 4 Wellness Center, 16 Philadelphia Ave, Shillington, PA 19607 USA; 5 Center of Excellence in Computational Molecular Biology, Faculty of Medicine, Chulalongkorn University, Bangkok, Thailand; 6 Department of Rehabilitation, Shanghai Children’s Hospital, School of Medicine, Shanghai Jiao Tong University, Shanghai, China; 7 Diagnosis and Treatment Center of Pitt-Hopkins Syndrome, Shanghai Children’s Hospital, School of Medicine, Shanghai Jiao Tong University, Shanghai, China

**Keywords:** Pitt–Hopkins syndrome, *TCF4*, Genotype, Phenotype, Developmental delay, Intellectual disability

## Abstract

**Background:**

Pitt–Hopkins syndrome (PTHS) is a neurodevelopmental disorder that remains underdiagnosed and its clinical presentations and mutation profiles in a diverse population are yet to be evaluated. This retrospective study aims to investigate the clinical and genetic characteristics of Chinese patients with PTHS.

**Methods:**

The clinical, biochemical, genetic, therapeutic, and follow-up data of 47 pediatric patients diagnosed with PTHS between 2018 and 2021 were retrospectively analyzed.

**Results:**

The Chinese PTHS patients presented with specific facial features and exhibited global developmental delay of wide severity range. The locus heterogeneity of the *TCF4* gene in the patients was highlighted, emphasizing the significance of genetic studies for accurate diagnosis, albeit no significant correlations between genotype and phenotype were observed in this cohort. The study also reports the outcomes of patients who underwent therapeutic interventions, such as ketogenic diets and biomedical interventions.

**Conclusions:**

The findings of this retrospective analysis expand the phenotypic and molecular spectra of PTHS patients. The study underscores the need for a long-term prospective follow-up study to assess potential therapeutic interventions.

**Supplementary Information:**

The online version contains supplementary material available at 10.1186/s13023-024-03055-7.

## Background

Pitt–Hopkins Syndrome (PTHS, OMIM #610954) is a rare neurodevelopmental disorder that was first described in 1978 [1]. The syndrome is characterized by early-onset developmental delay, moderate to severe intellectual disability (ID), specific facial features, and sometimes breathing anomalies [1, 2]. Haploinsufficiency for the TCF4 gene which encodes transcription factor 4 (TCF4, OMIM *602272) was discovered to be causative for the syndrome in 2007 [3, 4]. The TCF4 protein product contains a broadly expressed basic helix–loop–helix (bHLH) domain which is functionally characterized as DNA binding and known to be involved in embryologic neuronal differentiation [2, 3, 5, 6]. Although the pathogenic mechanisms are not fully understood, recent studies have suggested that TCF4 loss-of-function may impair Wnt signaling activity in neural progenitor cells, leading to reduced proliferation [7]. Moreover, by developing a novel TCF4 conditional mouse model and performing the proof-of-concept viral gene therapy experiments, the genetic normalization approach has been demonstrated as a treatment strategy for PTHS [8].

The prevalence of PTHS in diverse population has not been established yet [9]. Estimates from the United Kingdom and the Netherlands, based on the number of known affected individuals, suggest a prevalence range of 1 in 225,000 to 300,000 [2]. However, limited information is available regarding the prevalence of PTHS in China, and only a few Chinese PTHS cases have been reported [10, 11]. Due to the scarcity of data in the international literature and the heterogeneity in clinical presentation, molecular diagnostic criteria, and care practices, a group of international experts has promoted the first international consensus statement on the diagnosis and management of Pitt–Hopkins syndrome [2]. Currently, there is no cure for PTHS, and the clinical management primarily involves surveillance for common comorbidities and interventions targeting specific symptoms [2, 6].

Ongoing studies are focusing on identifying and targeting the molecular pathways affected by the TCF4 pathogenic variants [6, 7]. However, due to the incomplete underlying of the pathogenetic mechanisms and the clinical heterogeneity of symptoms, further research is needed to provide a comprehensive understanding of the mutational and clinical spectra of PTHS. These efforts could lead to better diagnosis, understanding of disease mechanisms, and the development of new treatment options [6, 11–18].

Herein, we present a cohort of 47 Chinese pediatric patients with PTHS, including 4 individuals previously described [11]. These patients were molecularly confirmed with pathogenic TCF4 variants, many of which were novel mutations. Frequencies of phenotypic traits and pathogenic variants observed in this Chinese cohort were compared with those reported in other populations. Additionally, genotype–phenotype correlation analysis was also performed. This study represents the first large-scale report of PTHS in the Chinese population.

## Materials and methods

Forty-seven patients, clinically diagnosed and molecularly confirmed with Pitt–Hopkins syndrome (PTHS) between 2018 and 2021, were retrospectively reviewed. The study was conducted at the Shanghai Children’s Hospital and approved by the Ethics Committee of the hospital. The requirement of written informed consent was waived by the ethics committee due to the retrospective nature of this study. Standardized assessments including GESELL, Griffith, and S–S (Sign–Significate relations), were employed to evaluate the developmental status of the patients. For assessing autism spectrum disorder (ASD), the ABC (Autism Behavior Checklist) and CARS (Childhood Autism Rating Scale) assessment tools were used. Patient information was tabulated without individual patient identifiers. Additionally, a score was assigned to each patient based on the clinical scoring rubrics proposed by Zollino et al. [2] to evaluate the sensitivity and specificity of the criteria. Related therapeutic interventions utilized were also discussed.

## Results

### Demographic data

A total of 47 patients, 29 males and 18 females, were included in this study (Table 1). The age at diagnosis varied from 6 months to 12.5 years with an average of 2.5 ± 2.3 years (mean ± standard deviation) and a median of 1.7 years. The average age at the time of the last physical examination was 3.5 ± 2.6 years (mean ± standard deviation), with a median of 3.0 years.

**Table 1:** Description of the 47 individuals with PTHS

	Number	Percent
Sex		
Male	29	62%
Female	18	38%
Age at last examination		
0–1 year	3	6%
1–3 years	20	43%
3–5 years	17	36%
5–10 years	5	11%
10–16 years	2	4%
First something was wrong		
During pregnancy	10	21%
Birth-6 months	21	45%
6 months–1 year	16	34%
Age at diagnosis	Mean 2.5 years, range: 6 months–12.5 years	

The parents of the patients were all unrelated and the majority of patients had no family history of developmental disabilities (except one with a family history of cerebral palsy). Abnormalities during pregnancy were observed in 26% (12/47) of the mothers. The most common abnormality was maternal hyperglycemia (7/47, 15%) (Table 2). Additionally, 11% (5/47) of the patients experienced hypoxia either at birth or during fetal development, and 6% (3/47) showed lateral ventricle enlargement detected through B scan ultrasonography during pregnancy.

**Table 2:** Abnormalities during pregnancy and neonatal period in the 47 PTHS patients

Abnormalities	Percent
Mother during pregnancy	
Hyperglycemia	7/47 (15%)
Hypothyroidism	3/47 (6%)
Abnormal amniotic fluid volume	2/47 (4%)
Threatened abortion	3/47 (6%)
Hypertension	2/47 (4%)
Fetus during pregnancy	
Lateral ventricle enlargement	3/47 (6%)
Abnormal fetal heart rate	3/47 (6%)
Fetal distress	2/47 (4%)
Intra-uterine growth retardation	1/47 (2%)
Situs inversus totalis	1/47 (2%)
Left renal polycystic dysplasia	1/47 (2%)
Fetus at birth	
Birth hypoxia	3/47 (6%)
Pathologic jaundice	4/47 (9%)
Hemolytic anemia due to G6PD deficiency	1/47 (2%)
Neonatal intracranial hemorrhage, Neonatal Septicemia	1/47 (2%)

## Clinical features

The majority of patients were initially identified to have developmental delay because milestones were not reached. Specifically, 5% (2/40) of the patients exhibited delayed head raising (> 6 months), 78% (32/41) experienced delayed unassisted sitting (> 9 months), and 93% (41/44) achieved independent walking at a later age (> 18 months). Among the 32 patients over 2 years old at their last medical visit, 59% (19/32) were able to walk, although most exhibited an unsteady gait starting at an average age of 5 years. However, only 16% (5/32) of patients developed speech, with abilities ranging from a few words to short sentences, starting at an average age of 2.6 years.

The clinical features of PTHS patients are summarized in Table 3. According to the latest proposed international clinical diagnostic scores [2], 64% (30/47) of the patients had a score of 9 or higher, representing clinically confirmed PTHS, while 28% (13/47) had a score of 6 to 8, indicating a suspicion of PTHS and a need for molecular testing. All 47 patients exhibited distinct facial characteristics and displayed varying degrees of developmental delay. Further details are shown in the SI (Additional file 1).

**Table 3:** Main clinical manifestations in 47 individuals with PTHS

Feature	Current study		Published study [2]		Other published study [19]	
	Number	Percent	Number	Percent	Number	Percent
Facial features						
Narrow forehead	4747	74%	127/150	85%	–	–
Square forehead	47/47	100%	–	–	–	–
Prominent forehead	42/47	89%	–	–	–	–
Thin lateral eyebrows	31/47	66%	110/149	74%	–	–
Wide nasal bridge/ridge/tip	44/47	94%	137/150	91%	44/45	98%
Flared nasal alae	46/47	98%	119/150	79%	27/47	57%
Full cheeks/prominent midface	47/47	100%	127/150	85%	36/48	77%
Wide mouth/full lips/cupid bow upper lip	47/47	100%	142/150	95%	37/48	79%
Thickened/overfolded helix	47/47	100%	96/141	68%	–	–
Short philtrum	34/47	72%	–	–		
Deep-set eyes	42/47	89%	–	–	23/41	56%
Up slanting palpebral fissures	43/47	91%	–	–	–	–
Widely spaced teeth	25/33	76%	–	–	–	–
Short neck	47/47	100%	–	–	–	–
Development						
Severe ID/GDD	42/44	95%	146/150	97%	48/48	100%
Very limited or absent speech	29/32	91%	135/150	90%	32/48	68%
Delayed gross motor development	47/47	100%	142/149	95%	44/48	94%
Gait ataxia	27/29	93%	88/128	69%	26/48	55%
Hypotonia	38/41	93%	104/142	73%	27/48	57%, > 90%[20]
Autonomic dysregulation						
Breathing anomalies	19/45	42%	81/145	56%	19/48	40%
Intermittent hyperventilation	17/45	38%	72/145	50%	–	–
Apnea	4/45	9%	61/145	42%	–	48%
Gastrointestinal abnormalities						
Constipation	31/47	66%	115/142	81%	40/48	85%
Food intolerances/allergy	17/21	81%	–	–	–	–
Sleep disturbances	2/27	7%	–	–	7/39	18% [6]
Neurological/behavioral features						
Microcephaly	3/47	6%	28/144	19%	84/144	59%
Brain MRI abnormalities	30/38	79%	–	–	–	60–70%[20]
Ventricle enlargement	17/38	45%	–	–	–	–
Wide extracranial space	13/38	34%	–	–	–	–
Myelination dysplasia/delay	7/38	18%	36/96	38%	–	–
Corpus callosum hypoplasia or agenesis	5/38	13%	–	–	–	–
Frontal lobe dysplasia	2/38	5%				
Electroencephalogram abnormalities	5/33	15%	–	–	–	–
Seizures	5/46	11%	55/1	37%	17/47	36%
Smiling appearance	31/34	91%	31/140	22%	82/94	97%
Anxiety/agitation	15/31	48%	–	–	32/67	48%
Autism spectrum disorder	10/15	67%				61%[18]
Regression	3/47	6%	6/150	4%	–	–
Visual anomalies	35/41	85%	–	–	25/48	53%
Myopia	22/34	65%	78/144	54%	110/207	54%
Strabismus	25/35	71%	70/144	49%	114/234	49%
Astigmatism	19/34	56%	45/144	31%	–	–
Hand features						
Slender fingers	47/47	100%	63/130	48%	–	–
Single transverse palmar crease	22/35	64%	60/120	50%	65/109	60%
Stereotypic hand movements	39/39	100%	87/146	60%	83/107	78%
Genitourinary features						
Small penis/ Cryptorchidism^a^	9/14	64%	–	–	–	33%

^a^Only male patients were counted

As evidenced in Table 3, a few patients in our cohort exhibited microcephaly, but most of their head circumference were relatively small, though within normal range. Notably, a significant proportion of patients displayed features such as a square forehead, prominent forehead, and full cheeks/prominent midface. Subtle abnormalities were detected in 79% of PTHS patients with brain magnetic resonance imaging (MRI) examinations. While electroencephalogram (EEG) results did not reveal any obvious abnormalities in the majority of patients (28/33, 85%), epileptiform discharges were identified in 3 patients and slow background activity in 2 patients. Given that most outpatient EEG sessions are typically short in duration and have a low positivity rate, long-term video EEG monitoring that covers the complete sleep cycle is necessary to detect electrical discharges during sleep.

Epilepsy was reported in 5 individuals and the 11% incidence was lower than the previously reported rate of 37–50% [19, 21]. In earlier studies, seizure onset appears to be highly variable raging from the first year of life to adolescence or early adulthood [2, 18]. The lower incidence observed in our study may be attributed to the age of our patient population. Since reported by the patient's parents, epileptic manifestations such as opisthotonos are easily detected, absence (petit mal) seizures, and petit seizures during sleep may be missed. Similarly, the age of onset of respiratory problems such as apnea also exhibits variability.

Stereotypic hand movements, characterized by repetitive flapping, twisting, waving, or flicking of hands and/or fingers, were observed in all patients. Among the 16 patients who underwent direct in-person assessment, 10 (67%) met the criteria for ASD, which is considered to be part of the classic PTHS phenotype [22, 23]. Constipation problems were common (66%) in the patients and they often present from infancy and last lifelong [19, 20]. Notably, five patients experienced a cessation of constipation issues (mean age of cessation: 2.5 years) with the introduction of complementary foods or dietary adjustments. Food intolerances/allergies were reported at a higher frequency (81%) compared to the general population, as previously described [2], with 17 out of 21 affected patients reporting intolerances to foods such as eggs, milk, soybeans, and wheat. Moreover, 33% of the patients were found to have elevated levels of immunoglobulins (IgE, IgM, and IgG). Regression of motor or speech abilities was noted in 3 out of 47 individuals. Two of these individuals had abnormal brain MRI findings, including corpus callosum hypoplasia and a wide extracranial space. The third individual had a combination of congenital heart disease and a family history of cerebral palsy, and experienced a seizure after a viral infection, which was followed by motor regression at the age of 1.5 years.

## Molecular findings

**Fig. 1:**
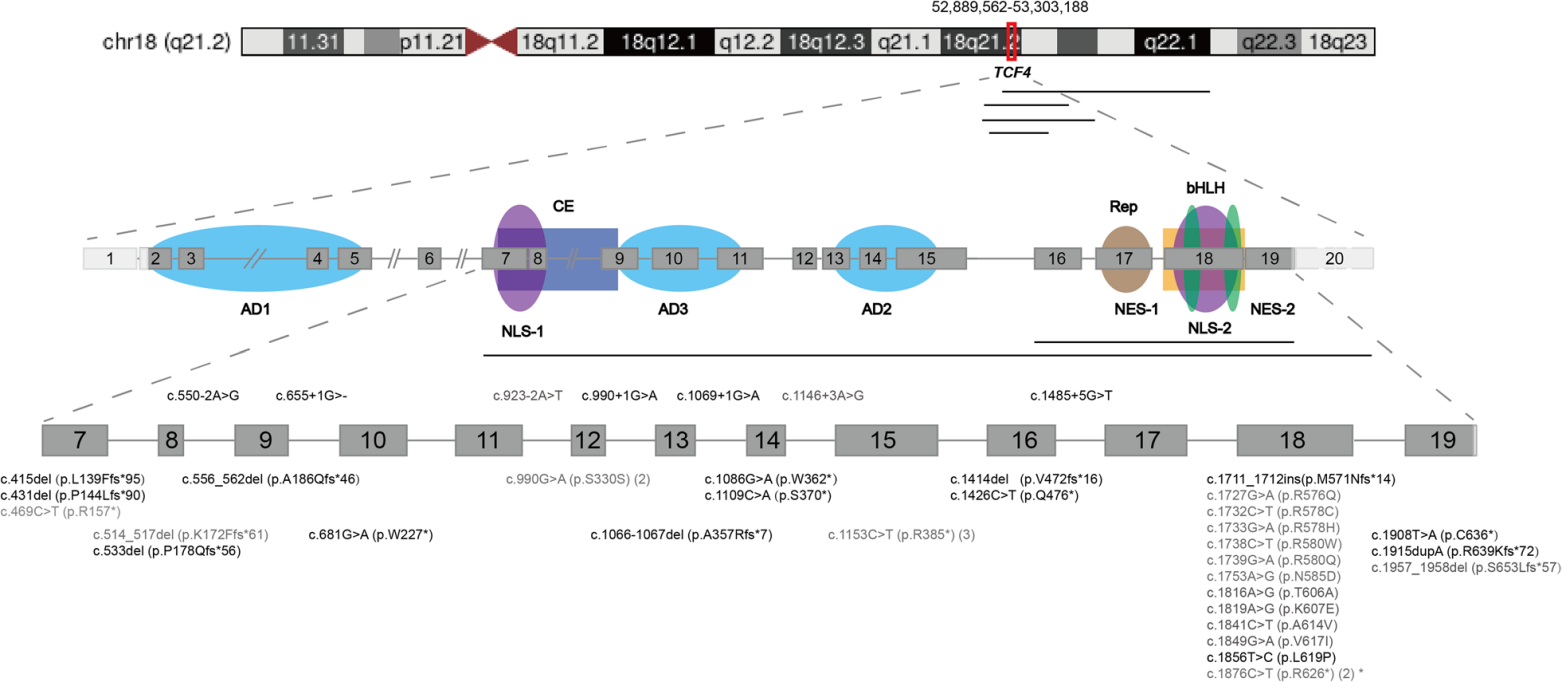
Schematic representation of the 47 reported Pitt–Hopkins patients disease-causing mutations. The colored shaded areas represent functional domains, including the AD activation domain, NLS nuclear localization signal, CE conserved element, Rep repression domain, NES nuclear export signal, and bHLH basic helix-loop-helix DNA-binding domain. The mutation coordinates are provided according to the TCF4 transcript variant 1 (NM_001083962.2) and GRCh37 for all probands. Horizontal black lines indicate deletions. The black color represents novel variants identified in this study, while the grey color represents variants identified in this study that have been reported previously. The asterisk * denotes a variant (c.1876C > T, p.R626) with a 20% degree of mosaicism identified in this study

Molecular analysis revealed that approximately 13% of patients in the cohort had copy number variants involving all or part of the TCF4 gene (Fig. 1). The remaining patients were identified to have pathogenic point mutations, including missense (11/47, 23%), nonsense (11/47, 23%), synonymous (2/47, 4%), and splice site (7/47, 15%) mutations, as well as small insertions or deletions leading to frameshift (10/47, 21%). Among these mutations, all were de novo mutations except in three cases the parental genetic information was not known. Notably, one variant (c.1876C > T, p.R626*) exhibited a 20% level of mosaicism. In particular, the two synonymous variants (c.990G > A, p.S330S), which alter the last nucleotide of exon 12, have been previously reported to modify the donor splice site [21, 24]. Additionally, the remaining two recurrently variants (c.1153C > T and c.1733G > A) have also been reported in previous studies [3, 24]. In total, 19 novel variants were identified. However, no clear genotype–phenotype correlations were observed in this study.

## Preliminary exploration of treatment

Symptomatic treatment is commonly administered to patients with PTHS. In our patient cohort, early intervention focused on rehabilitation training, including physical therapy, speech therapy, and occupational therapy, due to the high prevalence of early-onset developmental delay.

Patients presenting with epilepsy are typically treated with anti-seizure medications (ASMs), although the effectiveness may vary [18]. In our study, one patient exhibited a limited response to ASMs but showed significant improvement after initiating a ketogenic diet. The patient (c.923-2A > T), aged 7 years and 1-month, experienced frequent complex movements accompanied by autonomic symptoms. Video EEG revealed abnormal brain activity, and initial ASMs treatment did not yield satisfactory results due to gastrointestinal reactions. However, after three months of ketogenic diet therapy, seizure frequency was reduced by over 90%. This case suggests that ketogenic diet may be a viable alternative treatment for drug-resistant epilepsy in PTHS patients.

Eight patients in our cohort received biomedical interventions (Table 4), including an analysis of gut microbiota through stool samples. Seven children demonstrated yeast overgrowth, while only one child tested it was negative. Specific carbohydrates diet (SCD) and anti-candida supplements targeting digestive health were prescribed to children. Most children reported constipation prior to biomedical treatment. And after a few weeks of intervention, it has made significant improvements in digestive function, reduced constipation, and improved mental clarity in 7 children, except one child who was under 2 years old and did not respond well to special diet. Additionally, enhancements in speech, social communication ability, and emotional expression were noted. It is important to acknowledge that the diet employed in this study was relatively strict and challenging for most children to adhere to. Furthermore, the study period was limited to a maximum of three months.

**Table 4:** Eight patients received biomedical intervention in this study

PatientID	Sex	Age	Mutation	Phenotype	Intestinal flora test results	Gastrointestinal related symptoms (before the intervention)	Gastrointestinal related symptoms (after the intervention)
1	F	1y	(chr18: ?_52544772-58359295_?)*1 5.81 Mb deletion	Sitting unsteadily alone, no spontaneous speech, abnormal EEG	Yeast overgrowth	Constipation and food sensitivity	Little improvements
2	M	1y6m	c.990 + 1G > A	Support station, no spontaneous speech, normal EEG	Yeast overgrowth	Constipation, food sensitivity and digestive issues	Improvements in social skill and concentration
3	M	4y5m	c.1816A > G p.T606A	Walking at 17 m, passive calling at 3 y	Yeast overgrowth	Constipation, food sensitivity and mild case of autism	Improvements in many areas including speech, social skill and digestive system
4	M	5y	(Chr18:51,552,720–54,807,345)*1 3.25 Mb deletion	Standing unaided at 2 y, walking unaided at 4 y, no spontaneous speech, abnormal EEG	Yeast overgrowth	Constipation	No constipation. Improvements in immune system and speech
5	M	3y8m	c.1109C > A p.S370*	Walking at 2.2y, no spontaneous speech, poor cognition, salivation, and poor swallowing	Yeast overgrowth	Constipation, lower concentration and mild case of autism	No constipation. Concentration and expression in emotion and needs improved a lot, but still no speech
6	M	1y8m	c.655 + 1G > -	Support station at 1y8m, unconscious language and poor cognition. Born with Hypothyroidism	Yeast overgrowth	Constipation and a face as pale as wax	Almost no constipation. Facial appeared to be normal
7	M	1y5m	c.681G > A	Sitting at 11 months, stand at 16 months and able to call his mother consciously	Negative	Severe constipation, hyperactive and difficult concentration	Less constipation and much more calming
8	M	1y5m	c.1738C > T p.R580W	Sitting at 16 months, poor lateral support, no spontaneous speech, epilepsy	Yeast overgrowth	Constipation, indigestion, food sensitivity	Improvement in speech, motor skills and digestive system

*F* female, *M* male

## Discussion

The majority of patients with PTHS in this study exhibited early-onset severe motor delay and typical facial features. Language development was severely delayed, with most patients being non-verbal. Nonetheless, some individuals with milder symptoms have been identified [11, 13, 26]. Our findings provide a comprehensive description of clinical features that have not been systematically documented, including a square forehead, short philtrum, and abnormalities observed in brain MRI. Overall, the frequencies of commonly reported features in our patient cohort were similar to those previously published in the literature, except for a lower frequency of microcephaly, apnea, and seizures, as well as a higher frequency of gait ataxia in our cohort.

The typical facial gestalt has been the key feature for the diagnosis of PTHS [2, 20, 24], especially when distinguishing from other neurodevelopmental disorder such as Rett syndrome and Angelman syndrome. The overall sensitivity of the clinical diagnostic score system [2] is 91% and 4 patients with a score of 5 would be missed according to this set of criteria. All of them presented with typical facial features, but three patients were too young (average 0.73 years old) to assess other clinical information such as walking and speech and the other one (c.1849G > A, p.V617I) was the mildly affected. As we reported, relying solely on clinical criteria will lead to missed diagnoses. Thus, it is necessary to perform genetic testing early in diagnostic work-up, followed by standardized clinical assessment and treatment of symptoms [6]. With the wide-spread application of whole exome sequencing, the time to diagnosis has significantly decreased compared to before.

Molecular alterations responsible for PTHS in our cohort are as diverse as previously reported [2–4, 6, 21, 27]. Notably, these alterations are mainly located between exon 7 and 19 [2, 16]. Furthermore, all pathogenic missense variants (11/11) cluster in the highly conserved bHLH domain encoded by exon 18, which are predicted to have a variable structural impact by impairing protein’s DNA-binding, dimerization, transactivation ability and intranuclear localization [28]. Different mutations disrupt the transcription factor functions of TCF4 through different mechanisms and correspondingly contribute to phenotypic variability to some extent [25, 29]. However, patients with the same mutation (3 recurrently mutated variants c.990G > A, c.1153C > T, c.1733G > A) in our cohort presented with varying disease severity. As we have reported, the clinical presentation of patients is complex and can be influenced by other factors, such as timely intervention, abnormalities during pregnancy at birth and the presence of other genetic problems like G6PD deficiency.

The milder or more non-specific phenotype associated with those TCF4 variants is only partially explained so far [2]. Individuals with variants affecting exons 9–19 usually have typical PTHS [2, 12]. However, patient 35 (Additional file 1) is a 2.5-year-old girl with a milder phenotype, who has a genetic variant c.1849G > A (p.V617I) located in exon18 of TCF4, which we have previously reported [11]. She has a typical PTHS face and never demonstrated any abnormal breathing or seizure. Specifically, she obtained more advanced motor and verbal skills. She could ambulate independently and run normally but was unable to jump at 26 months. At 2 years old, she had a vocabulary of 20 words and occasionally used 2-word phrases. The mild clinical phenotype was thought that the V617I mutation induces subtle conformational changes compared with other missense mutations in exon18 affecting DNA binding directly or indirectly [11].

Individuals with variants in exons 1–4 and exon 4–6 were thought to have non-specific mild ID and not in the spectrum of PTHS [2, 12]. Here we report a case with mild ID and a deletion in chr18:53089161–53191438, which disrupted the exon4-5 in NM_001083962.2 of TCF4. The patient (patient 48 in Additional file 1) is a 13-years-old girl and exhibited mild dysmorphic facial features including flared nasal alae, full cheeks and cupid bow upper lip. She also had slender fingers and gait ataxia. As a result, she got a score of 6 according to the clinical diagnostic score system [2] Growth delay was noted when she was nine months old and she received rehabilitation treatment from nine months to five years of age. She raised head at 3 months, rolled over at 7 months, sat independently at 9 months and could walk unaided from 21 months of age, but with an unstable, ataxic gait. She developed some language from around 3 years and started to put together 4–5 words at age of 4.5 years. She never demonstrated any spells of abnormal breathing, but developed epilepsy one month after birth, which persisted until she was seven years old. During this time, she was treated with valproate, oxcarbazepine, and lamotrigine. Her full-scale intelligence quotient (IQ, WPPSI-IV) was 66 (mild) and met criteria for ASD. Currently, she had acquired self-care skills and enjoys singing, swimming, public speaking, and hosting a show. The mild phenotype may be attributed to mutations located in the 5′ region of the TCF4 gene, which only affect the expression of long TCF4 isoforms [24, 25], as well as the timely rehabilitation.

Individuals with variants affecting exons 7–8 were thought to present with moderate to severe ID and in the absence of the typical facial dimorphisms [2, 12]. However, the 5 patients with variants in exons 7 and 8 in the study all showed typical facial features (patient 1–5 in the Additional file 1). They have an average age of 3 years old, none of them showed symptoms of epilepsy, and only one had abnormal breathing. Among of them, patient 2 was a 2.8 girl with a variant c.431delC (p.P144Lfs*90) and had a moderate ID with a IQ of 43. She received rehabilitation training from 6 months and she is the only one who has so far shown any ability to speak. She started to speak the first word consciously around 30 months, which represented the emergence of autonomous language.

Early identification for neurodevelopmental disorders enables timely intervention for affected individuals, thus affecting their subsequent development. While gene abnormalities or congenital brain lesions are contributing factors, environment and experience also play a role in brain development. Research has shown that early intervention in infants biologically at risk of developmental disorders, regardless of the presence of a brain lesion, is associated with improved cognitive development in early childhood without affecting motor development [30]. The theory of brain plasticity and functional reorganization supports the idea that earlier intervention leads to better outcomes. Early identification also allows for better management of the disease, identification of potential therapeutics, and avoidance of unnecessary treatments for affected individuals. Additionally, accurate diagnosis can relieve emotional distress for families and help them to evaluate family planning options.

The ketogenic diet is an important treatment option for refractory epilepsy, including cases caused by gene mutations, including rare ones [31–33]. Current studies suggest that the antiepileptic effects of the ketogenic diet are mediated by ketone bodies, which affect neurotransmitters, brain energy metabolism, oxidative stress, and ion channels. In our study, one patient with medically intractable epilepsy attempted ketogenic therapy and experienced a positive effect. However, the limited sample sizes and generally short-term follow-up resulted in low- to very low-certainty evidence for the outcomes.

Preclinical studies have demonstrated the mechanisms by which the microbiome influences bidirectional gut–brain communication [34]. In our study, biomedical intervention also conducted to find a correlation between specific micro bacteria and Pitt–Hopkins Syndrome. From our findings, it appears that yeast overgrowth may be connected to TCF4 gene mutation, although this is only a conjecture. However, this gives us a promising direction for further research.

As this is a retrospective study, the clinical data, including cognitive, behavioral, and neuropsychological profiles are incomplete and only clinical features up to the date of last visit are available. To explore genotype–phenotype correlations and potential therapeutic interventions further, a long-term prospective follow-up cohort is necessary for further studies.

## Conclusions

We summarized the clinical phenotypic and molecular data of 47 Chinese pediatric cases with PTHS and expanded the data spectrum of PTHS patients. Three cases of related therapeutic interventions, including ketogenic diets and biomedical interventions showed promising results. A long-term follow-up and a larger sample size would be required in future studies.

### Supplementary Information

**Additional file 1: Table S1**. A table detailing the clinical signs and genomic defects.

## Data Availability

The data presented in this study are available on request. The data are not publicly available due to privacy restrictions.

## Abbreviations

PTHS: Pitt–Hopkins syndrome
ID: Intellectual disability
TCF4: Transcription factor 4
bHLH: Basic helix–loop–helix
MRI: Magnetic resonance imaging
ASMs: Anti-seizure medications
ASD: Autism spectrum disorder
SCD: Specific carbohydrates diet
